# Drug Target Prediction and Repositioning Using an Integrated Network-Based Approach

**DOI:** 10.1371/journal.pone.0060618

**Published:** 2013-04-04

**Authors:** Dorothea Emig, Alexander Ivliev, Olga Pustovalova, Lee Lancashire, Svetlana Bureeva, Yuri Nikolsky, Marina Bessarabova

**Affiliations:** IP & Science, Thomson Reuters, Carlsbad, California, United States of America; Institute for Research in Biomedicine, Spain

## Abstract

The discovery of novel drug targets is a significant challenge in drug development. Although the human genome comprises approximately 30,000 genes, proteins encoded by fewer than 400 are used as drug targets in the treatment of diseases. Therefore, novel drug targets are extremely valuable as the source for first in class drugs. On the other hand, many of the currently known drug targets are functionally pleiotropic and involved in multiple pathologies. Several of them are exploited for treating multiple diseases, which highlights the need for methods to reliably reposition drug targets to new indications. Network-based methods have been successfully applied to prioritize novel disease-associated genes. In recent years, several such algorithms have been developed, some focusing on local network properties only, and others taking the complete network topology into account. Common to all approaches is the understanding that novel disease-associated candidates are in close overall proximity to known disease genes. However, the relevance of these methods to the prediction of novel drug targets has not yet been assessed. Here, we present a network-based approach for the prediction of drug targets for a given disease. The method allows both repositioning drug targets known for other diseases to the given disease and the prediction of unexploited drug targets which are not used for treatment of any disease. Our approach takes as input a disease gene expression signature and a high-quality interaction network and outputs a prioritized list of drug targets. We demonstrate the high performance of our method and highlight the usefulness of the predictions in three case studies. We present novel drug targets for scleroderma and different types of cancer with their underlying biological processes. Furthermore, we demonstrate the ability of our method to identify non-suspected repositioning candidates using diabetes type 1 as an example.

## Introduction

Finding novel ways to treat and cure diseases is a fundamental challenge in biomedical research. Although many advances have been made over the last decades, drug discovery is still a very lengthy, increasingly risky and costly process [1]. There is a lack of reliable drug target prediction methods as reflected by the low clinical target validation success rate. Therefore, new bioinformatics approaches are required, which are able to accurately predict drug targets for a disease [2]. These predicted drug targets can be of two types. 1. *Novel drug targets*: unexploited targets that can be used for developing first in class drugs and combination therapies. 2. *Drug targets for repositioning*: drug targets that are currently used in the treatment of a different disease. Many targets are functionally important and are pleiotropically involved in multiple pathologies [3], [4]. As pathologies are often shared between diseases, the existing or experimental drugs against these targets can be re-tested for such additional indications [5].

Over the last years, various network-based methods have been developed for identification of unknown disease-associated genes [6]. There is evidence that these methods may be applied to the prediction of novel drug targets as disease-associated genes and successful drug targets significantly overlap [7]. However, the actual performance of network-based methods for drug target prediction has not been comprehensively assessed to date. Early approaches for disease gene prioritization incorporated knowledge about disease linkage intervals with protein interaction networks and prioritized direct interactors of known disease genes [8]–[10]. Following these methods, research was focused on integrating additional *local* information to the prioritization by exploiting the network neighborhood of known disease genes. Dezso and colleagues, for example, prioritized disease-associated candidates by their presence on shortest paths between known disease genes [11]. Other approaches identified modules that are differentially regulated in the disease of interest: Ideker and colleagues first developed a method to identify subnetworks that exhibit distinct regulation patterns across different biological conditions [12]. A following study by Ulitsky *et al* built on this idea, resulting in a method for unraveling dys-regulated pathways in a disease of interest [13]. Recent approaches improved the previous ones even further by incorporating the complete *global* network topology into the disease gene prioritization. Koehler and colleagues applied random walks to predict novel disease-associated candidates assuming that genes in close overall proximity to known disease genes are more likely to be involved in the disease themselves [14]. Finally, Vanunu *et al* developed network propagation, a flow-based method similar to random walks that prioritizes genes by their proximity to all known disease genes [15]. Global methods generally perform better than local and module-based methods. However, a recent study by Navlakha and Kingsford highlighted that the integration of predictions from global and local methods outperforms the results from each method since each method captures specific network features and thus uniquely prioritizes certain disease genes [16].

**Figure 1:**
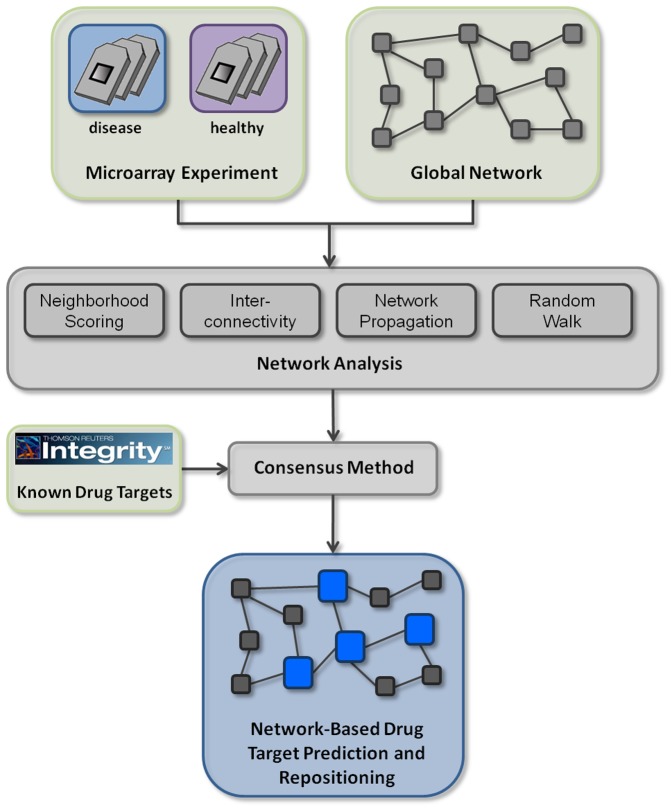
Overview of the workflow. The analysis starts with a set of microarray samples from diseased and healthy donors, which is statistically processed to identify differentially expressed genes (DEGs). Furthermore, a high-quality interaction network serves as input to the analysis. The DEGs are overlaid onto the network and serve as input to the four network analysis methods, namely Neighborhood Scoring, Interconnectivity, Network Propagation, and Random Walk. The output of the methods is aggregated using a logistic regression model, which is trained on a set of drug targets from Integrity, resulting in the final ranked list of prioritized gene products.

In this study, we developed an integrated network-based approach that enables both the prediction of novel drug targets and the repositioning of known drug targets for a given disease (Figure 1). Our method takes as input a gene expression signature for a disease of interest as a source of disease-specific information. Gene expression patterns systematically change in response to the disease, which is evident from thousands of studies and datasets deposited in the GEO repository [17]. Thanks to well-established microarray technology, global expression profiles are probably the most readily available and the richest source of disease expression data, applicable for different purposes. Connectivity Map, for instance, pioneered drug repositioning by comparing drug response expression with disease expression signatures [18], [19]. Expression profiling can also be integrated with knowledge-based information such as molecular interaction networks, enhancing the latter with disease and tissue context [20]. Network-based methods imply that drug targets are highly influential in establishing a disease-specific expression response and likely correspond to expression regulators [21], [22]. Therefore, it is logical to use differential gene expression profiles as input for the prioritization of potential drug targets. We hypothesize that drug targets, while not necessarily dys-regulated themselves, are located in close overall proximity to the differentially expressed genes, which can be assessed using established network-based methods. In our approach, the differentially expressed genes are overlaid onto a high-quality molecular interaction network. The drug target prediction for a disease is performed by applying a number of local and global network-based prioritization methods using expression signature genes as an input. The predictions from these methods are combined using a logistic regression model resulting in a set of prioritized drug targets for the disease. The prioritized drug targets can serve as candidates in the development of novel drugs for a disease. Furthermore, if the drug target is already used for a different indication, it can be readily evaluated as a candidate for the disease of interest.

We demonstrate that our approach is able to reliably predict known drug targets. The performance evaluation was done for 30 different diseases based on information about known drug targets for the diseases. Here, we provide prioritized lists of predicted drug targets for all of these 30 indications as a source of data for further discovery of novel drug targets and drug target repositioning. In addition, top candidate targets for several indications were analyzed in more detail and underlying potential mechanisms of action were suggested. We first studied a novel drug target candidate for scleroderma in detail and unraveled the underlying biological processes involved in the disease. Furthermore, we identified a common core of cancer drug targets that are associated with a multitude of cancer types and that may inhibit core functionalities of cancer cells. We additionally analyzed highly ranked drug targets that are specific to a certain type of cancer only and that may thus lead to selective treatment options. Finally, we demonstrate the ability of our method to identify promising candidates for drug target repositioning using diabetes type 1 as case study. Since our method does not rely on disease similarities for prioritizing repositioning candidates, connections between seemingly unrelated diseases can be identified leading to the emergence of non-suspected drug target candidates.

## Materials and Methods

### Disease Gene Expression Signatures

We obtained microarray gene expression data for 30 diseases from the Gene Expression Omnibus (GEO) repository [23]. The experiments were required to contain samples from healthy and diseased patients (see Table 1 for the complete list of diseases). The expression data were normalized with the GC-RMA package implemented in R. Gene level estimates were obtained using custom CDF files downloaded from the brainarray database [24]. For each gene expression dataset, disease samples were compared to samples from healthy donors. Significance p-values for all gene expression changes between the samples were calculated using a moderated t-test with FDR correction contained in the R limma package [25]. A gene was defined as differentially expressed between diseased and healthy subgroups if its fold change was greater than 1.5 and if the FDR corrected p-value was less than 0.05.

**Table 1:** Overview of diseases in the study.

Disease Name	GEO Accession	Number of DEGs	Number of Drug Targets
Malaria	GSE5418	1658	220
Acute myeloid leukemia	GSE30029	5325	159
AIDS	GSE16363	1839	40
Idiopathic pulmonary fibrosis	GSE24206	14	31
Thyroid carcinoma	GSE29265	3979	37
Colorectal cancer	GSE35602	2952	304
Crohn's disease	GSE10714	12	75
Diabetes type 1	GSE11907	471	68
Hepatitis C	GSE11907	2626	229
Hepatocellular carcinoma	GSE36411	2425	210
HIV	GSE18233	463	159
Hyperplastic polyposis syndrome	GSE19963	897	3
Ischemic cardiomyopathy	GSE5406	146	5
Ischemic stroke	GSE16561	290	322
Liver cirrhosis	GSE36411	121	10
Melanoma	GSE15605	4038	175
Melanoma with metastasis	GSE15605	5469	73
Multiple sclerosis	GSE32988	428	379
Obesity	GSE12050	1631	245
Ovarian cancer	GSE38666	3862	15
Parkinson's disease	GSE22491	1142	347
Periodontitis	GSE10334	1416	28
Psoriasis	GSE26866	310	351
Sarcoidosis	GSE34608	5803	13
Scleroderma	GSE33463	232	11
Septic shock	GSE26440	3455	131
Sickle-cell disease	GSE16728	559	4
Sjogren's syndrome	GSE23117	271	8
Systemic Lupus Erythematosus	GSE11907	476	62
Ulcerative colitis	GSE10714	871	57

For each disease, the table lists the GEO accession for the gene expression data sets, the number of differentially expressed genes (DEGs), and the number of drug targets associated to the disease in Integrity. The number of DEGs and drug targets are based on Entrez Gene identifiers.

### Integrity Drug Targets

Integrity (http://integrity.thomson-pharma.com) is a knowledgebase designed for drug discovery. The database contains a large collection of drugs which are annotated with information on their respective drug targets, the diseases they are associated with, and the clinical phases of the drugs.

Drug targets are assigned a status in Integrity, which can be ‘Validated’, ‘Candidate’, ‘Exploratory’, or none. Validated drug targets are associated with drugs under active development in clinical phases or with launched drugs for the disease of interest. Candidate drug targets are associated with drugs that are no longer under active development for the respective disease. Exploratory drug targets are associated with drugs that are currently under biological investigation for the disease. Finally, some drug targets are not assigned any status and were not considered in this study. For each disease used in our analysis, we downloaded its associated drug targets. In Integrity, drugs are not directly linked to genes. Instead, drugs are linked to internal target IDs and these targets are then linked to Entrez Gene identifiers. Here, the Entrez Gene – drug target associations were considered as true positives for each disease and were used to evaluate the drug target predictions.

### Selection of Diseases

We selected 30 diseases based on several criteria: 1. We aimed at selecting a variety of diseases to demonstrate the broad applicability of our method. The diseases range from cancers to metabolic diseases to viral infections. 2. To calculate disease gene expression signatures, availability of healthy and diseased samples was required for this study. 3. We only considered significant differentially expressed genes, i.e. genes that passed an FDR-corrected p-value threshold of 0.05. At least one differentially expressed gene is necessary as input for the network-based methods. 4. For the logistic regression model to work, at least two drug targets are required as true positives. Therefore, diseases were required to be associated with at least two drug targets in Integrity.

### Molecular Interaction Network

We employed the MetaBase resource to build the network used in this study [26]. In MetaBase, all molecules are stored as *network objects*. Network objects describe the type of molecule, e.g. kinases, transcription factors, and receptors. Network objects may also correspond to more than one biological molecule such as a protein complex or a protein family. Furthermore, network objects can represent small molecules including non-coding RNAs and human metabolites.

The interactions contained in MetaBase represent physical interactions between pairs of network objects and have all been manually curated from publications of small-scale experimental studies. These interactions include mostly protein interactions and regulatory interactions between transcription factors and their targets, but also a limited number of interactions involving non-coding RNAs and metabolites. The interactions are annotated with additional information including directionality and mechanism of action, i.e. activation, inhibition, and unknown effects. Furthermore, MetaBase stores information on linear pathways, describing the cellular response to a particular stimulus. Linear pathways are defined based on manual curation and depict the signaling cascade from receptor activation through the cell to the final response.

To build the interaction network, we integrated all interactions with known mechanism of action with the interactions contained in the linear pathways, resulting in 115,781 non-redundant high-confidence interactions between a total of 19,130 network objects.

### Network-Based Methods

Network-based methods can generally be grouped into local and global methods. Local methods make use of the neighborhood of disease-associated genes to prioritize novel candidates. Global methods take the whole network and its topology into account to identify new disease-associated candidates. In this study, we apply two local and two global methods for the prediction of drug targets, namely Neighborhood Scoring, Interconnectivity, Network Propagation, and Random Walks. The input to all four methods is the list of differentially expressed genes for the diseases of interest.

#### Neighborhood Scoring

Neighborhood Scoring is a local method for prioritizing candidates based on the distribution of differentially expressed genes in the network [27]. We adapted the method such that every network object is assigned a score, which is based partly on its expression fold change and partly on the expression fold changes of its neighbors. First, the differential expression levels of the genes are mapped to the corresponding network objects. Next, an adjusted differential expression level, the score, is calculated for each network object as follows:
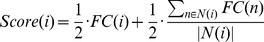



The score of network object *i* equally depends on its fold change (*FC*) and on the fold changes of its neighbors *n*, where *N(i)* includes all neighboring network objects of *i*. Network objects that are not differentially expressed and that do not have any differentially expressed genes in their direct neighborhood are assigned a score of 0.

#### Interconnectivity

Interconnectivity is a local method that prioritizes candidates based on their overall connectivity to the differentially expressed genes [28]. First, an interconnectivity score is calculated for each pair of interacting network objects. The interconnectivity score is based on both the direct interaction between a pair and the indirect interactions with a path length of two, which we define as the shared neighborhood of two network objects. We adapted the method to score interactors of differentially expressed genes based on their direct interaction and on their shared neighborhood as follows:
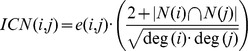

*e(i,j)* describes an edge between the two network objects *i* and *j*. It is set to 1 if the edge exists and 0 else. Besides the direct interaction between *i* and *j*, the size of their shared neighborhood *N* is taken into account and normalized by the overall degrees of the two network objects.

Next, each network object receives its final score based on the interconnectivity to all differentially expressed genes:
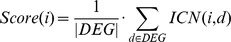
where *d* represents a differentially expressed gene and *DEG* the set of all.

#### Random Walk

A random walk describes the transition of a random walker through a network [14]. It is regarded a global method because the complete network structure is exploited in these walks. In a random walk, a set of starting points in the network is defined, corresponding to the differentially expressed genes here. In each iteration, the random paths are extended by transitioning to an adjacent network object with equal probability. Additionally, a random walk has a certain probability of terminating and restarting from the starting points. In each step, the network objects are assigned probabilities describing the chance of a random walk traversing this object. Upon convergence of the probabilities, the network objects are ranked by their visitation probabilities. Network objects with high probability scores are most proximal to all starting points and are considered candidates:
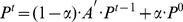

*P^t^* is a vector containing the visitation probabilities for all network objects at time point *t*. *A^’^* describes the normalized adjacency matrix of the network, which has been transformed into a stochastic matrix. *P^0^* represents the vector of starting points for the random walk, where each network object corresponding to a differentially expressed gene is assigned the same starting probability. Finally, α is a weighting factor, assigning a certain probability for the random walk to continue and for a restart from the starting points.

#### Network Propagation

Network Propagation is a global method that takes the complete network topology into account for prioritizing candidates [15]. Network Propagation is similar to Random Walks in thought. First, the differentially expressed genes are mapped to the corresponding network objects. Each of these objects is assigned a score of 1, while the remaining network objects are assigned a score of 0. These scores represent the prior knowledge of the disease and are smoothed over the network to prioritize candidates that are in close proximity to all differentially expressed genes.

The scoring of the network objects can be regarded as propagating flow through the network. The starting points of the flow are the differentially expressed genes and in each iteration, the flow is further pumped through the network until a steady state is reached. The final flow that each network object received corresponds to its final score and defines the rank of the object in the list of candidates. In each iteration, the flow for the network objects is updated as follows:
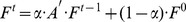

*F^t^* is a vector containing the flow for each network object at time point *t*. A′ corresponds to the adjacency matrix of the graph, where each entry is normalized by the degrees of the source and target nodes. The normalization by node degrees compensates for the fact that nodes with many interactors have a higher chance of picking up flow by chance and are thus more likely to be ranked higher in the prioritization. *F^0^* represents the prior knowledge vector containing the scores for differentially expressed genes. The algorithm terminates when the L_1_ norm of the difference between *F^t^* and *F^t-1^* drops below 10^−6^.

### Consensus Method and Performance Evaluation

Each of the network-based methods results in a ranked list of all network objects producing four lists per disease. As shown previously, combining predictions from multiple methods improves the overall predictive power [16]. Therefore, we built a consensus method using a logistic regression model that was trained on the true positive drug targets from Integrity. The model integrates the predictions from the four methods and results in a final list of prioritized network objects for each disease. Analyses were implemented in the R caret package [29].

In more detail, for each disease D, we built a matrix containing the scores for each network object assigned by the four methods, i.e. each row corresponds to a network object, and each column corresponds to one method. Then, we added an additional column to the matrix representing the output vector, which indicates whether the network object is a known drug target for disease D or not. If it is a known target, the column entry is 1 ( =  positive) and 0 ( =  negative) otherwise. This matrix served as input for training a logistic regression model for disease D.

Using a combined approach of model selection and performance evaluation we obtained disease-specific regression models. Precisely, we used 5-fold cross-validation as follows: we split the input matrix and took 80% of the network objects as training set and the remaining 20% as test set. We repeated this partitioning step five times, i.e. always taking a different 80% and 20% such that every network object occurs in a test set exactly once. For each training set, a regression model was built and used to make predictions for the left out test set. The model for each training set was optimized using a bootstrapping procedure for parameter tuning. Finally, the predictions made for all test sets were aggregated, resulting in our final prediction list for disease D.

Since the datasets are very imbalanced in terms of positives and negatives, random partitioning into training and test sets in the 5-fold cross-validation would not be able to retain the original balance between the positive and negative samples as found in the complete dataset. Therefore, our modeling procedure includes a constraint in the partitioning step, i.e. the step where we define the training and test sets, known as stratified cross-validation. Here, the class proportions for each fold are as close as possible to the class proportions of the entire data set, thus maintaining the original balance.

For each disease, the overall performance of the predictions was assessed with a Receiver Operating Characteristics (ROC) curve, using the Integrity drug targets for disease D as true positives and the remainder treated as negatives.

### Permutation Test to Assess Baseline Performance

We used a permutation test to assess the baseline performance for each disease. If the input disease gene signature is *independent* of the known drug targets, the performance of a random regression model will be similar to the real disease model, because random input genes will return comparably good results. On the other hand, if the input disease gene signature and the known drug targets are *dependent*, the regression model output will be better for the real data than for the permuted.

For the permutation test, we kept the input matrix for disease D, i.e. the rows correspond to the network objects and the columns to the features. Now, we randomly permuted the assignment of positives and negatives, i.e. the assignment of known drug targets for the disease, and re-ran the complete modeling procedure. Each permutation test resulted in an AUC value, which may be greater than 50% in an unbalanced setting. For each disease, we performed the permutation test 100 times and calculated the median of the resulting AUCs to obtain the baseline estimate for each disease.

### Clustering of Diseases

Disease similarity was analyzed at two levels: at the level of gene expression signatures and at the level of predicted drug targets. At the level of gene expression signatures, we calculated the distance matrix for the disease pairs based on the overlap between sets of differentially expressed genes using the Jaccard coefficient as a measure of the overlap. The same approach was used for calculating the distance matrix at the level of predicted dug targets, where the top 100 predicted drug targets for each disease were used for the calculation.

Next, the diseases were clustered using hierarchical clustering with complete linkage. We used the Mantel test to assess the similarity between the gene signature-based distance matrices and the predicted drug target-based distance matrices [30]. The Mantel test calculates the correlation between two matrices, where the p-value is a departure from zero correlation over 1000 permutations of the rows and columns.

## Results and Discussion

### Drug Target Prediction and Repositioning Workflow

Here, we suggest an analysis workflow for drug target prediction and repositioning for a disease of interest based on network analysis of a disease-specific gene expression signature (Figure 1). The majority of known drug targets are not differentially expressed themselves in a disease and thus cannot be identified from the disease gene expression signature directly. Analyzing the 30 indications used throughout this study revealed that the fraction of targets that are differentially expressed in a disease varied between 0% and 42% (data not shown). However, drug targets should be influential on the disease gene expression and thus in close network proximity to differentially expressed genes.

We applied the workflow to 30 different diseases, ranging from cancers to viral infections and metabolic diseases. Unlike other diseases, viral infections are currently treated by targeting viral proteins. However, the ability of viruses to quickly acquire resistance mutations demands for exploring novel treatment strategies. Therefore, large-scale RNA interference screens have been performed to identify human host factors. Host factors are essential for virus replication but are not lethal to human cells when knocked down and can thus serve as potential new drug targets [31]–[34]. We collected gene expression data for the diseases from GEO and obtained a gene expression signature for each disease as a set of genes that are differentially expressed in diseased patients (Table 1). Each signature was overlaid onto a high-quality molecular interaction network as an input to the candidate prioritization methods, namely Neighborhood Scoring, Interconnectivity, Random Walk and Network Propagation. Each of these network-based prioritization methods resulted in a unique prioritized list of candidate network objects. To aggregate these lists, we built a consensus method using a logistic regression model, trained on a set of manually annotated known drug targets from the Integrity database, which led to an integrated list of prioritized network objects (Table 1). These known drug targets are not necessarily targeted by approved drugs, but by drugs at any developmental stage. Although chances are high that early stage drug targets eventually fail in clinical trials, such failures are usually due to unwanted side effects and not due to biological relatedness to the disease. Therefore, even early stage targets can be invaluable for better understanding the biological mechanisms of a disease.

According to our hypothesis, the prioritization of network objects is directly related to their likelihood of being a drug target. Correspondingly, we considered the higher scored network objects as potential drug targets for a given disease. The top drug target prediction for each disease is shown in Table 2, and the complete list of predictions can be found in Table S1.

**Table 2:** Top drug target predictions for the 30 diseases.

Disease Name	Top Drug Target Prediction	AUC	Baseline AUC
Hyperplastic polyposis syndrome	SP1	93.19	69.83
Periodontitis	Atp6v1c2	90.98	54.27
Scleroderma	STAT1	90.39	55.48
Idiopathic pulmonary fibrosis	PTPR-beta	88.26	53.34
Thyroid carcinoma	c-Myc	85.67	54.03
AIDS	LSm complex	84.94	53.29
Ischemic cardiomyopathy	eIF1AY	84.92	58.14
Ovarian cancer	Intelectin-1	83.83	57.33
Sickle-cell disease	Dynactin complex	82.07	64.72
Acute myeloid leukemia	SP1	81.95	51.62
Melanoma	c-Myc	81.63	51.75
Sjogren's syndrome	P53	81.44	57.71
Colorectal cancer	c-Myc	81.13	51.08
Hepatocellular carcinoma	P53	80.19	51.88
Psoriasis	5T4	79.25	50.94
Melanoma with metastasis	c-Myc	78.79	52.55
Septic shock	c-Jun	78.68	52.32
HIV	STAT1	77.64	51.33
Obesity	SP1	76.65	51.48
Malaria	c-Myc	75.79	51.33
Systemic lupus erythematosus	STAT1	75.28	53.03
Parkinson's disease	Olfactory receptor	75.16	51.35
Sarcoidosis	c-Myc	74.06	56.15
Diabetes type 1	EGR2 (Krox20)	72.89	52.58
Hepatitis C	c-Myc	72.15	51.46
Ulcerative colitis	c-Jun	71.74	52.14
Multiple sclerosis	P53	71.51	50.94
Crohns disease	VPS52	68.44	52.19
Liver cirrhosis	STAT1	67.47	54.85
Ischemic stroke	CLEC2D	63.27	51.18

For each disease, the name of the predicted drug target obtained from MetaBase and the AUC performance together with the respective baseline AUC performance (based on permutation testing) is shown.

Investigating the importance of each of the four network methods for building the logistic regression model, we observed that for the majority of diseases, the global and local methods equally contribute to the predictions. For some diseases the global methods dominate the predictions, while the predictions for few other diseases mainly rely on the local network methods. Table 3 summarizes the importance of each method for the predictions in each disease. Overall, Network Propagation contributes the most information for the majority of diseases, followed by Interconnectivity. Random Walk and Neighborhood Scoring only rank first in few diseases but as can be seen from the table, they do contribute some information to the model. These findings highlight the necessity of integrating diverse methods into the prioritization process as they all add valuable information to the predictions.

**Table 3:** Overview of network analysis method importance.

Disease Name	Network Propagation	Random Walk	Interconn-ectivity	Neighborhood Scoring
Periodontitis	**100**	45.7	15	3.7
Thyroid carcinoma	**88.8**	47.2	83.2	0
Scleroderma	60.5	0	**100**	32
Idiopathic pulmonary fibrosis	0	0	**100**	1.9
AIDS	58.4	**100**	38.4	0
Ischemic cardiomyopathy	0	20.8	14.4	**100**
Liver cirrhosis	34.9	0	**100**	0
Melanoma with metastasis	**100**	26.2	80	0
Ovarian cancer	82.1	35.4	**87.2**	0
Sjogren's syndrome	**84.8**	47.6	69.6	0
Sickle-cell disease	**100**	0	30	1
Hyperplastic polyposis syndrome	**96.8**	0	63.2	0
Colorectal cancer	88.6	19.1	**100**	0
Acute myeloid leukemia	89	34.1	**100**	0
HIV	64.2	21.9	**100**	0
Hepatocellular carcinoma	**99.5**	41.2	88.3	0
Sarcoidosis	**80**	74.1	0	61.4
Psoriasis	**100**	31.7	51.1	0
Melanoma	**96.6**	14.7	96	0
Obesity	**100**	36.6	69.7	0
Septic shock	**88.1**	22.1	70.6	0
Malaria	76.5	21	**100**	0
Diabetes type 1	**92**	61.3	75.9	0
Multiple sclerosis	**100**	0	58.1	0.1
Systemic Lupus Erythematosus	66.6	27.4	**100**	0
Parkinson's disease	**100**	39.6	46	0
Ulcerative colitis	**92.1**	20	53.1	0.1
Hepatitis C	**94.9**	58.4	20	17.6
Crohns disease	0	0	**100**	1.9
Ischemic stroke	**100**	52.1	48.1	0

For each disease, the importance of the four network analysis methods for the consensus method is shown. The importance for each method ranges from very important (100) to not important (0). The most informative feature for each disease is highlighted.

### Performance Evaluation of Drug Target Predictions

The drug target predictions made for each disease were assessed using Receiver Operating Characteristics (ROC) plots. Here, the true positive rate is plotted against the false positive rate at varying thresholds. The performance of the predictor is measured by the Area Under the Curve (AUC).

**Figure 2:**
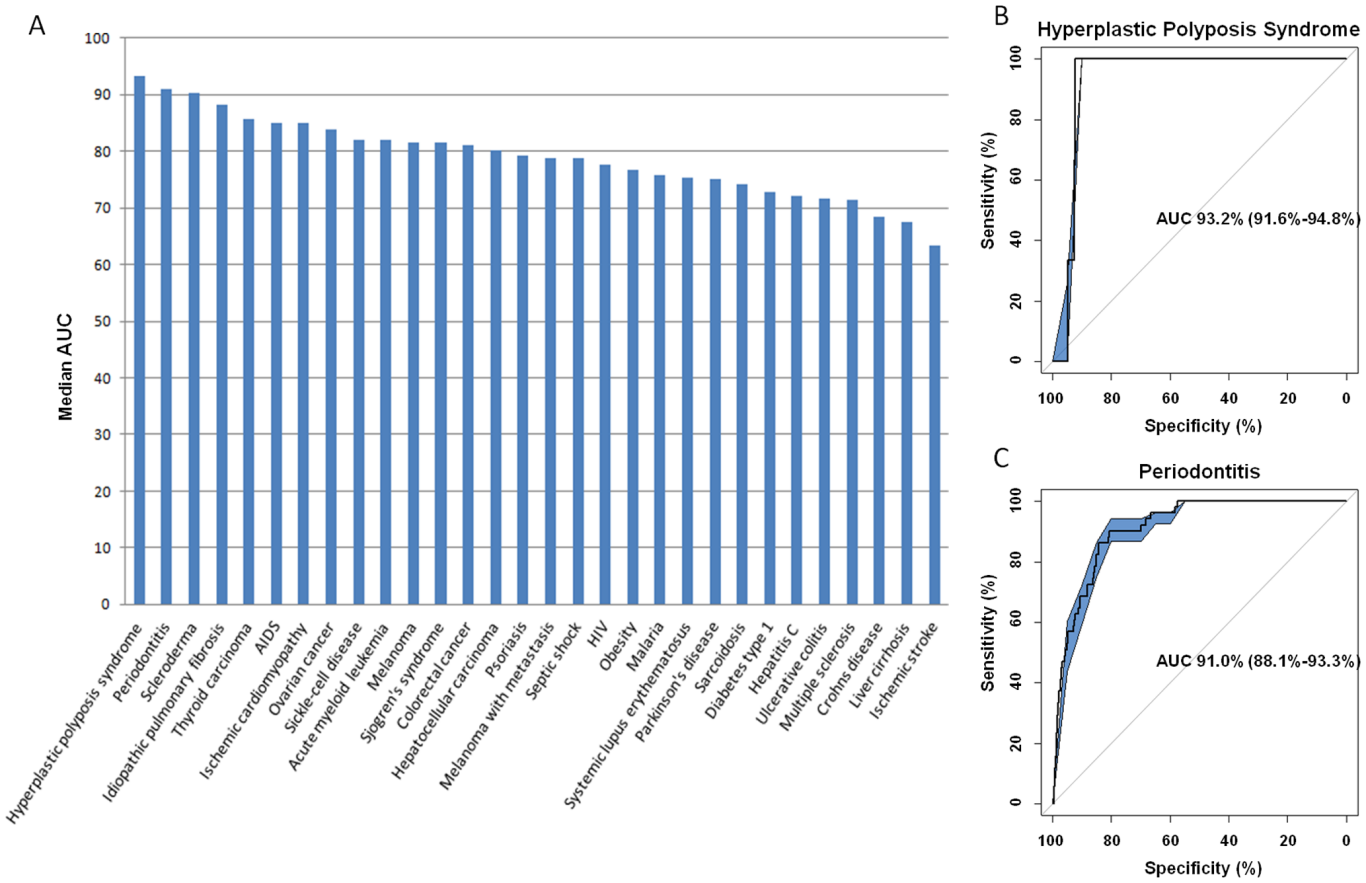
Consensus method performance. (A) The plot shows the median AUC for each disease model. The highest AUC of 93.19% is achieved for hyperplastic polyposis syndrome and the lowest for ischemic stroke with 63.27%. (B) and (C) show the ROC curves for hyperplastic polyposis syndrome and periodontitis, which achieved the highest performance. The blue areas around the AUC curves represent the 95% confidence intervals.

In order to evaluate the predictions, a set of true positives needs to be defined. Here, we treated the Integrity drug targets as true positives and all other network objects as negatives, although some of these negatives might in fact be currently unknown drug targets for the disease. As shown in Figure 2, the highest AUC was achieved for the disease hyperplastic polyposis syndrome with a median AUC of 93.19% closely followed by periodontitis with a median AUC of 90.98% (the ROC plots with confidence intervals for all 30 diseases are shown in File S1). The lowest performance was obtained for ischemic stroke with a median AUC of 63.27%, which is still well above a random prediction. For 14 diseases the AUC was above 80%; 13 other diseases had a demonstrated AUC higher than 70% and only 3 diseases had an AUC below 70%. The top-performing disease, hyperplastic polyposis syndrome, is associated with the fewest drug targets, which might lead to the impression that fewer targets automatically result in better AUC performance. However, liver cirrhosis, for example, has only 10 drug targets assigned but exhibits prediction performance below 70% (Table 2). Correlating the number of drug targets with the resulting AUCs resulted in a Pearson correlation of around −0.4, demonstrating that the performance is not dependent on the number of drug targets assigned.

However, since our datasets are highly imbalanced, i.e. the number of positives is much smaller than the number of negatives, the baseline AUCs may be above 50%. Therefore, we used a permutation test to compare the AUCs of the *real* prediction performance to the corresponding *baseline* performance obtained from the permutation test. As shown in Table 2, our method results in much higher prediction performance than expected by random chance. For most diseases, the baseline predictions result in AUCs slightly above 50%. However, the best-performing disease, hyperplastic polyposis syndrome, exhibits a baseline AUC close to 70%, likely a result of the particularly low number of known drug targets associated with the disease. This demonstrates that the baseline performance needs to be taken into account when working with imbalanced datasets. Here, the baseline provides additional confidence that the regression model performance is improved by using meaningful disease-specific gene signatures and not negatively affected by the imbalanced data.

As demonstrated, the performance of the drug target predictions varies between the diseases, though all of them are well above the baseline performance. The observed variability is likely due to a number of factors. For instance, the number of genes in the disease gene signatures varies, with acute myeloid leukemia and metastatic melanoma containing more than 5,000 genes and Crohn's disease and idiopathic pulmonary fibrosis containing less than 15 genes. Theoretically, a single gene in the disease gene signature is sufficient for the network-based methods to work. However, whether this number has an influence on the performance has never been assessed. Furthermore, the number of known drug targets differs for the diseases, ranging from more than 300 to less than 5, with a minimum of two drug targets required per disease. Additional factors such as the network topology, the location of the differentially expressed genes in the network, and their degree distribution may all contribute to the performance variability. Finally, the incompleteness of biological networks in general can have an influence on the performance of network-based methods [35]. Global methods are likely more robust to missing edges than local methods (as long as the network remains a large connected component) as they exploit all paths available in the network. However, the influence on performance may also vary between diseases, since some diseases tend to be more local than others. Table 3 shows that for some diseases the local methods outperform the global methods and thus the robustness may depend on the disease characteristics.

### Analysis of Drug Target Predictions

Among the drug targets predicted for a certain disease there are both completely novel drug targets, which have not been used to treat any disease to date, and drug targets that may already be used in the treatment of another disease. The first category of predicted drug targets can be used for the development of innovative therapeutic approaches, whereas the second class of drug targets allows repositioning the corresponding drugs to the disease of interest. Having proven that our network analysis method is able to predict drug targets known for the disease of interest with high accuracy for a wide range of diseases, we suggest that this methodology can be applied for both the prediction of novel drug targets and repositioning.

**Figure 3:**
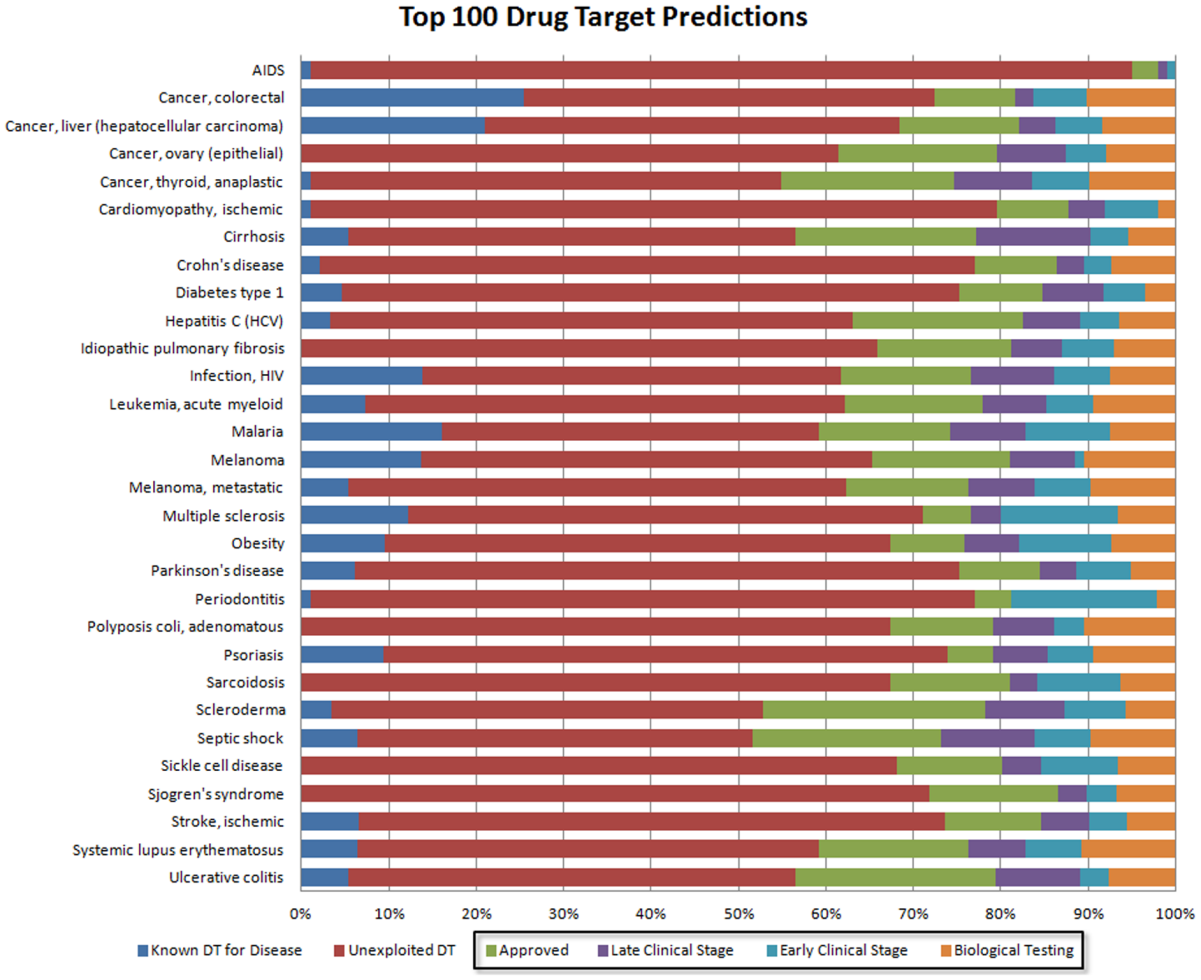
Analysis of top 100 drug target predictions. Blue represents the number of known drug targets for the disease. Drug targets that are currently not used to treat any disease are shown in red. The remainder represents drug targets that are used to treat other indications (highlighted by a black box). These drug targets are grouped into approved drugs, late stage clinical phases, early clinical phases, and biological testing.

Figure 3 shows the distribution of the different types of drug targets in the top 100 drug target predictions for each of the 30 diseases. Based on the annotations from the Integrity knowledgebase, we categorized drug targets as either approved, in late clinical stages, in early clinical stages, or in biological testing. Interestingly, for most diseases, approximately half of the top drug target predictions are unexploited, leading to potentially new treatment strategies. The other half, however, contains both known drug targets and a number of drug targets currently used to treat other indications. Most importantly, for all diseases, we predicted several drug targets that are already approved for a different disease as well as some drug targets that are in late clinical stages. Such drug targets can be readily repositioned for the treatment of a disease of interest. The classification of the top 100 predicted drug targets for all diseases is provided in Table S2.

### Predicted Drug Targets are Disease-Specific

The fact that the network analysis methods are able to predict known drug targets for a particular disease with high performance suggests that this analysis approach provides as an output unknown disease-specific drug target candidates. To further investigate the disease specificity of the predicted drug targets we clustered the 14 analyzed diseases with an AUC above 80% based on their overlap of differentially expressed genes as well as based on the overlap between their top drug target predictions. Selecting only those diseases with high predictive power assured that the clustering results were not affected by noise but reflected biological results. Diseases that share a high number of differentially expressed genes have similar underlying pathology and pathways involved. Therefore, if the network analysis methods predictions are disease-specific, these diseases are generally expected to exhibit similar patterns of drug target predictions. Computing the correlation between the distance matrices obtained from gene expression signatures and drug target predictions, we found a significant correlation between differential expression and target based clustering (p-value 0.008), indicating that similar disease gene signatures lead to similar drug target predictions. We also performed this analysis for the complete list of diseases and found a significant similarity between the distance matrices obtained from gene expression signatures and drug target predictions as well.

**Figure 4:**
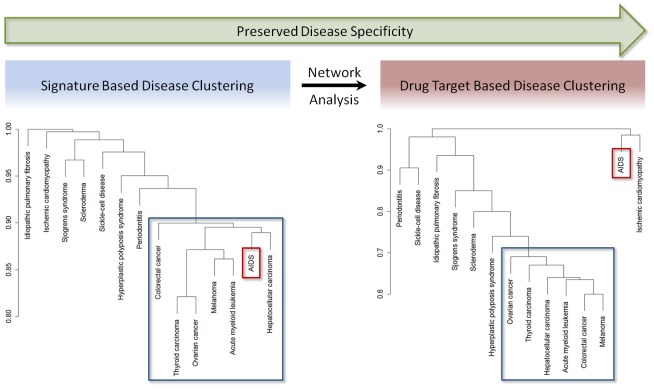
Clustering of 14 diseases. The clustering based on the gene expression signatures is shown on the left and the clustering based on the top 100 drug target predictions to the right. Cancer-related clusters are highlighted in blue and the placement of AIDS in the two clusterings is highlighted in red.

As shown in Figure 4, diseases with higher overlap of differentially expressed genes tend to result in more similar drug target predictions. Cancer-related diseases, for instance, can be grouped both at the level of differentially expressed genes and their predicted drug targets. Interestingly, clustering at the level of differentially expressed genes placed the melanoma samples more distantly from the other types of cancer, and put AIDS into the group of cancer-related diseases. On the level of drug targets, however, all cancer-related diseases cluster closely together, while AIDS is removed from this group of diseases. Therefore, although diseases may appear to be similar at the level of differentially expressed genes, their regulators and the biological processes involved in the disease may be distinct. Using the results of the drug target based clustering, we are thus able to identify diseases with unique or common underlying biological processes. Furthermore, the results of the analysis provide additional evidence of the disease-specificity of drug target candidates predicted based on our network analysis.

### STAT1 as a Novel Drug Target for Scleroderma

Scleroderma is a systemic autoimmune disease, which manifests in the remodeling of connective tissue and fibrosis [36], [37]. Current therapeutic approaches aim at decreasing both inflammatory processes and fibrosis [38]. According to the Integrity knowledgebase, 83 drugs are currently under development for the treatment of scleroderma and its symptoms, 35 of which have already been approved or are in clinical trials. Depending on their biological targets, the drugs are classified as stem cell therapy, apoptosis inducers, angiogenesis modulators, and immunosuppressants. The small number of approved drugs and the limited success of current treatment options, however, clearly demonstrate the need for developing new treatment strategies.

As described previously, we obtained the disease-specific gene expression signature for scleroderma from diseased and healthy peripheral blood mononuclear cells (PBMCs) (GEO accession GSE33463). PBMCs mainly include monocytes and lymphocytes, which are important components of the immune system. It has been demonstrated previously that monocytes from scleroderma patients can stimulate proliferation of fibroblasts and thus exhibit direct involvement in scleroderma-related fibrosis [39].

We first applied a pathway enrichment analysis to the dys-regulated genes in order to identify physiological processes related to the disease. Using the GeneGo Pathway Maps ontology we found a significant enrichment of signaling pathways related to immune responses [26]. In particular, the pathway “antiviral actions of interferons” was the most enriched pathway and “IFN alpha/beta signaling pathway” was found within the top ten. The importance of interferon signaling for the development of scleroderma has been suggested previously as interferon signaling may contribute to the progression of different autoimmune diseases [40]–[42].

Our drug target prediction identified the transcription factor STAT1 as the most promising drug target for scleroderma. According to the Integrity knowledgebase, STAT1 is not used as a drug target to date and may thus be exploited for developing novel treatment strategies. Interestingly, according to the gene expression signature, STAT1 is up-regulated in scleroderma. Furthermore, STAT1 is a key participant in interferon signaling [43], [44], which we identified as affected pathway in the enrichment analysis of differentially expressed genes.

**Figure 5:**
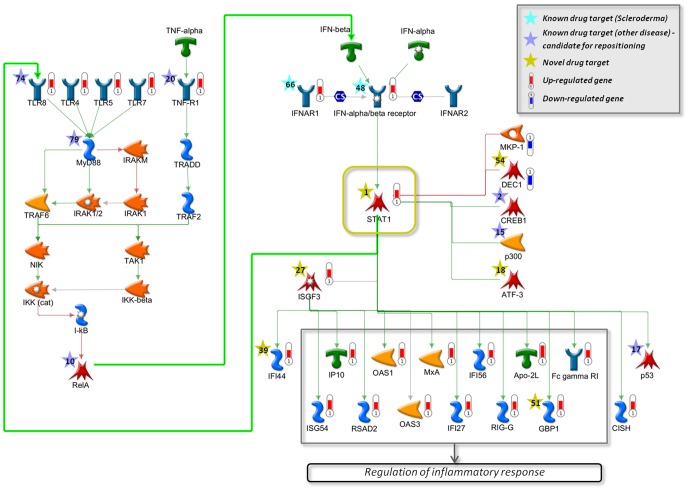
Network reconstruction for STAT1 signaling in scleroderma. TLR signaling is activated by STAT1, which in turn activates IFN signaling, resulting in increased STAT1 activity. Predicted drug targets (within the top 100) for scleroderma are highlighted with colored stars, where the numbers correspond to the rank in the drug target predictions. Cyan stars represent known drug targets for scleroderma. Purple stars correspond to drug targets that have been associated with other diseases and can be readily repositioned to the treatment of scleroderma, while yellow stars indicate unexploited drug targets that can be used for the development of novel treatment strategies. Red thermometers show significantly up-regulated genes in scleroderma, blue thermometers show down-regulated genes. Green arrows correspond to activation edges, red arrows represent inhibition edges.

To demonstrate the signaling mechanism, which relates STAT1 to scleroderma-specific differentially expressed genes and scleroderma pathogenesis, we reconstructed a molecular network around STAT1 (Figure 5). The network is composed of canonical signaling pathways that are enriched with predicted drug targets for scleroderma and scleroderma-specific differentially expressed genes. Firstly, STAT1 is a transcription regulator of the majority of these differentially expressed genes. Also, as shown in the network, STAT1 activates TLR signaling, which results in the activation of IFN signaling. STAT1 in turn is activated by the IFN-alpha/beta receptor, which is triggered by IFN-alpha and IFN-beta. According to the Integrity knowledgebase, the IFN-alpha/beta receptor is a known drug target for scleroderma, and in our drug target predictions, the receptor was identified as potential drug target within the top 50 predictions. TNF-alpha signaling, which is functionally related to and cross-talking with the IFN pathway, was also included in the network. Strikingly, besides the top drug target prediction STAT1, 14 of the network objects in this reconstructed network were predicted within the top 100 in our analysis, with eight of them within the top 20 predictions. Seven of the predicted drug targets, namely TLR8, TNF-R1, MyD88, RelA, CREB1, p300, and p53, are already known drug targets for other diseases and can thus be readily repositioned to the treatment of scleroderma. The remaining top drug target predictions, DEC1, ATF-3, ISGF3, IFI44, and GBP1, have not been associated with any disease yet and may be exploited for drug development for scleroderma in the future.

### A Common Core of Drug Targets for Cancers

**Figure 6:**
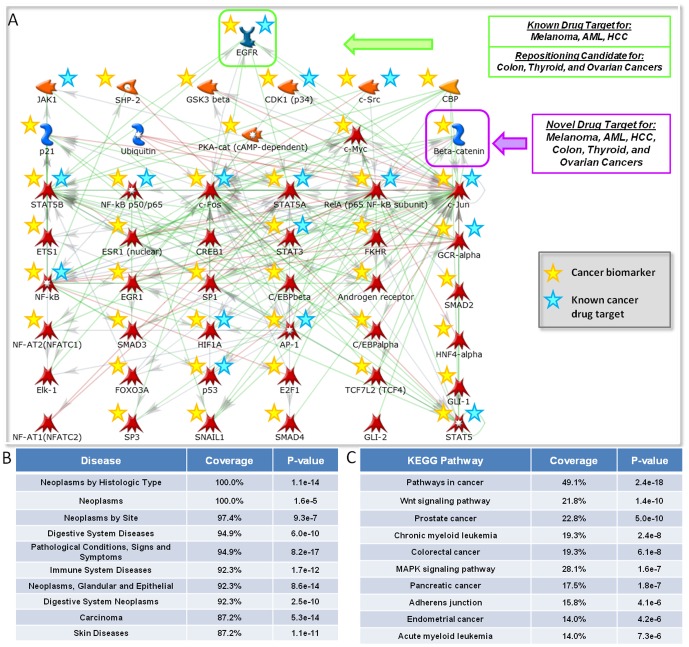
Core network of predicted drug targets in cancers. (A) shows the commonly predicted drug targets (within the top 100 predictions) for colorectal cancer, thyroid cancer, ovarian cancer, melanoma, acute myeloid leukemia, and hepatocellular carcinoma. Yellow stars represent known disease biomarkers for neoplasms obtained from the Metabase resource. Cyan stars highlight genes that are known drug targets for at least one of the six types of cancer. (B) shows diseases that are significantly associated with the predicted drug targets. The diseases are ordered by the percentage of genes they cover. Neoplasms are found to cover all of the predicted drug targets. (C) shows the most enriched KEGG pathways for the predicted drug targets [45]. Cancer-related pathways are most enriched followed by pathways for specific cancers as well as cancer-related signaling pathways.

**Figure 7:**
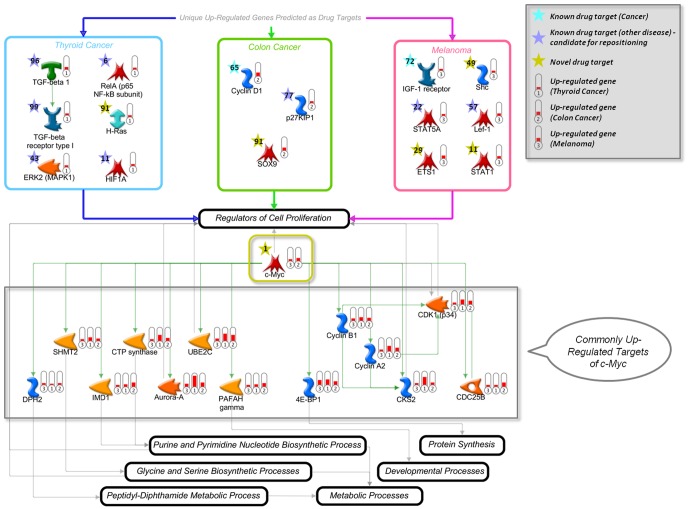
Network reconstruction for c-Myc as a common drug target in different cancers. The blue, green and magenta boxes show uniquely up-regulated genes that were predicted as drug targets (within the top 100 predictions) for the indicated cancer type and that contribute to the regulation of cell proliferation. c-Myc (in the middle) is the top drug target prediction for all three cancer types and is involved in the regulation of cell proliferation as well. Downstream targets of c-Myc are shown in the gray box below c-Myc and are uniformly up-regulated in all three cancer types. Cyan stars represent known drug targets for the respective cancer type. Purple stars correspond to drug targets that have been associated with other diseases and can be readily repositioned to the treatment of this type of cancer, while yellow stars indicate unexploited drug targets that can be used for the development of novel treatment strategies. Red thermometers show significantly up-regulated genes in (1) Thyroid Cancer, (2) Colon Cancer, and (3) Melanoma.

As discussed previously, the close clustering of different cancer types implies similarity of the underlying pathological processes, which may allow for the repositioning of drug targets between different cancer types. The prediction of similar drug targets for different types of cancers further led to the identification of a common core of drug targets shared by a multitude of cancers. Figure 6 shows a network of 48 predicted drug targets that have been predicted within the top 100 for thyroid cancer, colon cancer, ovarian cancer, melanoma, acute myeloid leukemia, and hepatocellular carcinoma. A disease biomarker enrichment analysis revealed that the predicted drug targets are mostly enriched in neoplasms and related diseases. Furthermore, the drug targets are found to be highly enriched in cancer-specific and cancer-related signaling pathways obtained from the KEGG database [45]. According to the Metabase resource, most of these predicted drug targets, 42 out of the 48 genes, are known biomarkers for the disease category of neoplasms and 17 of them correspond to known drug targets for at least one of the six cancer types mentioned previously (Table S3). Drug targets that have already been associated with some type of cancer can readily be repositioned to the treatment of other cancers, while novel drug target predictions may be exploited for new treatment options in a multitude of cancers.

c-Myc was predicted as the number one drug target for colorectal cancer, thyroid cancer, and melanoma (Table 2). According to the Integrity knowledgebase, c-Myc is not used as a drug target and may thus lead to novel treatment options for multiple cancers. The reconstructed c-Myc centered network reveals that c-Myc serves as a common endpoint of cancer regulatory cascades leading to the regulation of cell proliferation, which is the basic pathological process in cancer (Figure 7). Downstream targets of c-Myc likewise regulate proliferation and in addition metabolic processes, which are also considered prominent in cancer pathogenesis. The downstream targets are up-regulated in all three cancer types and therefore, targeting c-Myc may lead to therapeutic approaches that can be applied to a variety of cancers.

Considering that c-Myc is the topmost drug target prediction and that its downstream targets are consistently dys-regulated, c-Myc seems to be a promising target for therapy of the three cancer types. Indeed, c-Myc plays a well-known role in cancer pathology and it has been discussed as a potential target for anticancer therapy for more than a decade [46]–[48]. Promising applications of anti-c-Myc treatment resulted in tumor regression in vitro and in animal models [49]–[51]. However, when selecting c-Myc as a target for anti-cancer therapy, two important points should be considered. First, c-Myc induces numerous signaling pathways, including but not limited to tumor suppressing pathways [46], [52]. Therefore, the activity of all c-Myc induced pathways and cross-talking pathways need to be taken into account in their complexity to ensure responsiveness to the treatment. Second, c-Myc inhibition can have toxic side effects since it is ubiquitously expressed and exhibits activity in both normal and cancer tissues. Toxicity in particular is a serious issue for the majority of existing anti-cancer therapies and not specific to c-Myc treatment. A possible strategy to reduce the toxic side effects of c-Myc inhibition is the limited exposure to c-Myc inhibitors, which could be still beneficial for highly responsive patients identified based on patient stratification. Although such strategies have not yet been developed, promising results have been demonstrated in animal models of c-Myc-induced tumor genesis, which show tumor regression even after brief exposure to c-Myc inactivation [51].

Besides the common target, c-Myc, each cancer also involves unique regulatory mechanisms of cell proliferation. Targeting of these genes might thus lead to more specific treatment options for a particular cancer type. We selected these unique drug targets for each cancer type based on several criteria: the targets should be up-regulated in the respective cancer type, they should be positive regulators of cell proliferation, and they should be contained within the top 100 drug target predictions. For each of the three cancer types, we identified a number of genes that are already known drug targets for other diseases, such as TGF-beta 1 for thyroid cancer, p27KIP1 for colon cancer, and STAT5A for melanoma. Since drugs are already known for these genes, they may be readily repositioned for treatment of the respective cancer types. Furthermore, several of these genes correspond to currently unexploited drug targets, namely H-Ras, SOX9, Shc, ETS1, and STAT1, potentially leading to novel treatment strategies.

### COX-2 as a Repositioning Candidate for Treatment of Diabetes Type 1

COX-2 is an inducible enzyme participating in the production of prostaglandins, which in turn function as regulators of various immunological processes. Prominent COX-2 inhibitors include ibuprofen and aspirin and are commonly used for the treatment of arthritis and pain, but have also been explored for cancer treatment. Interestingly, our method predicts COX-2 with high confidence as drug target candidate for diabetes type 1. It ranks at position 6 in the prioritized list of network objects and represents the topmost candidate for drug target repositioning (Table S2). However, COX-2 has not been suggested as potential drug target for diabetes type 1 to date and it does not represent an obvious repositioning candidate as the current indications appear unrelated to diabetes type 1.

**Figure 8:**
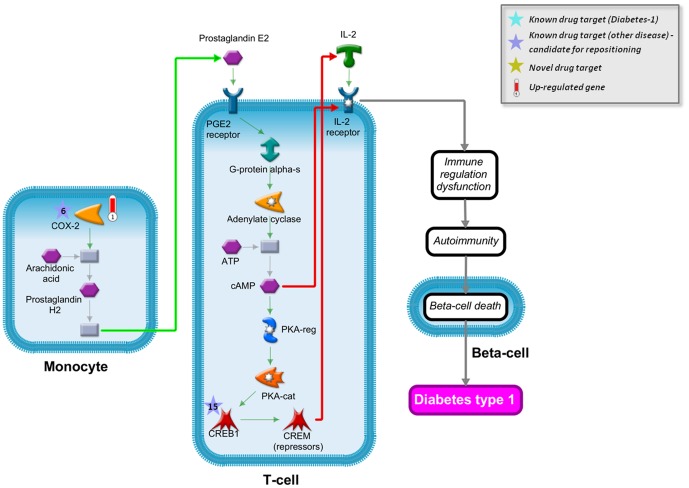
Network reconstruction for COX-2 as repositioning candidate for diabetes type 1 therapy. Over-expression of COX-2 in monocytes leads to an increased production of prostaglandin E2. Prostaglandin E2 activates T-cell signaling through the PGE2 receptor resulting in increased cAMP levels and activation of the transcription factors CREB1 and CREM. cAMP inactivates the IL-2 receptor of T-cells, while CREM acts as repressor for IL-2. The inhibition of IL-2 and the IL-2 receptor result in immune regulation dysfunction leading to autoimmunity and ultimately the death of beta-cells, which is the cause of diabetes type 1. Predicted drug targets (within the top 100) for diabetes are highlighted with colored stars, where the numbers correspond to the rank in the drug target predictions. Purple stars correspond to drug targets that have been associated with other diseases and can be readily repositioned to the treatment of diabetes type 1. Red thermometers show significantly up-regulated genes in diabetes type 1. Green arrows correspond to activation edges, red arrows represent inhibition edges.

Diabetes type 1 is characterized by progressive failure of insulin producing beta-cells caused by the development of autoimmune responses directed to beta-cells. The modulation of immune processes and inflammation is considered a potential target for treatment and prevention of insulin dependent diabetes [53], [54]. It has been demonstrated previously that COX-2 is over-expressed in monocytes of patients with diabetes type 1, which we also observe as shown in Figure 8. COX-2 over-expression suppresses the expression of interleukin-2 (IL-2) and its receptor in T-cells, which is expected to disrupt normal regulation of T-cells in immune responses [55]. As depicted in Figure 8, the mechanism can be mediated through the production of prostaglandin E2, for instance, which suppresses the production of IL-2 and the expression of the IL-2 receptor, resulting in the suppression of IL-2 signaling [56]-[58]. Disruption of the IL-2 pathway plays a significant role in the development of autoimmunity and insulin-dependent diabetes [59]. Moreover, COX-2 inhibitors demonstrated good results in the prevention of insulin-dependent diabetes development in preliminary experimental studies in laboratory animals [60]. Therefore, we believe that COX-2 may be a promising repositioning candidate for diabetes type 1 therapy with a number of approved drugs readily available.

## Conclusions

We developed a novel computational approach for drug target prediction and repositioning starting from sets of disease-specific differentially expressed genes as the molecular manifestation of pathology. The majority of known drug targets are not differentially expressed in the disease themselves and selecting candidates from the disease gene expression signature is thus a limiting factor. Our method, however, is capable of identifying candidate targets independent of their direct dys-regulation in the disease. We suggest calculating putative targets as regulators of the disease expression patterns and ranking them based on network proximity to disease DEGs. The proximity was computed by a set of local and global network analysis algorithms. The predictions made by individual algorithms were combined using a logistic regression model trained on the true positive drug targets from Integrity. The approach was evaluated using a comprehensive set of manually curated drug targets for 30 diseases and demonstrated high performance with AUCs ranging between 63.27% and 93.19% for different diseases.

Grouping of predicted drug targets reflects mechanistic anchoring for disease pathology. In one case study, several high-scoring predicted targets for scleroderma formed a subnetwork consisting of IFN and TLR signaling pathways and centered around the transcription factor STAT1. The network was highly enriched with known targets for scleroderma, which validates testing of other components of this mechanism, i.e. the predicted targets, as novel targets and candidates for repositioning. In a second case study, we compared sets of predicted targets for melanoma, thyroid and colon cancers. The targets formed a common core around c-Myc signaling with additional unique modules in each cancer. Both common and unique signaling contributed to regulation of proliferation. Finally, we were able to show the potential of our method for repositioning drug targets using diabetes type 1 as an example. While the repositioning candidate, COX-2, is a well-known target for pain and inflammation treatment, these diseases are not related to diabetes type 1 and the repositioning of COX-2 is thus unsuspected. Since our method prioritizes candidates based on the disease gene expression signature only and does not take potential disease similarities into account, truly novel target – disease associations can emerge.

Additional refinements may improve our target prediction method. For instance, drug targets may be filtered for unspecific candidates. The transcription factor SP1, for example, is highly ranked in a variety of diseases. SP1 regulates a large number of genes and is known to be involved in many cellular processes, including cell differentiation, cell growth, apoptosis, immune responses, response to DNA damage, and chromatin remodeling. Naturally, this makes SP1 a good candidate for diseases in general, but is not specific to a particular disease. Based on such biological knowledge, drug target predictions can be further limited to the most disease-specific candidates only. Besides filtering the final candidate lists, the construction of context-specific networks will allow for prioritizing those network objects that are actually present and interacting in a diseased cell. Our method is also readily extendible for both novel proximity algorithms and other types of experimental data, for instance whole genome sequencing.

The fact that drug targets are not necessarily differentially expressed in the corresponding diseases raises questions about the applicability of expression profiling alone in drug discovery. Our approach helps to overcome this drawback and is well applicable for both patient stratification in clinical trials and personalized treatment. In the former case, the patient cohorts can be defined based on similarity of sets of predicted targets, rather than on global expression profiles similarity. In personalized care, expression, genetic and other OMICs profiles from individual biopsies can be used as inputs for optimal target identification. Certainly, both hypotheses have to be thoroughly tested in clinical trials.

## Supporting Information

File S1 **Prediction performance for 30 diseases.** The file contains the ROC plots for all 30 diseases. The blue area around each curve represents the 95% confidence interval.
(DOC)

Table S1 **List of predicted drug targets for 30 diseases.** The table contains the prioritized list of network objects for each disease. Each network object is listed with the name as contained in the Metabase resource and its prediction score. Furthermore, each network object is annotated with drug target information from Integrity, where known drug targets for a given disease are marked with an x.
(ZIP)

Table S2 **Top 100 drug targets for 30 diseases.** The table contains annotations for the top 100 drug target predictions for all 30 diseases. Each network object corresponds to either a known drug targets for the given disease, a known drug target for other diseases, or no current drug target. Furthermore, network objects with increased expression levels are highlighted as they may be inhibited for treatment.
(XLS)

Table S3 **Commonly predicted cancer drug targets.** The table lists all network objects that were commonly predicted as cancer drug targets within the top 100 for six different cancer types. For each network object, the table highlights the types of cancer for which the network object is a known drug target. Furthermore, network objects corresponding to known cancer biomarkers are emphasized.
(XLS)
