# Supplementary material for: Drug Target Prediction and Repositioning Using an Integrated Network-Based Approach
Source: PLoS One. 2013 Apr 4;8(4):e60618. doi: 10.1371/journal.pone.0060618 (PMC3617101; doi:10.1371/journal.pone.0060618)

# Drug Target Prediction and Repositioning using an Integrated Network-Based Approach

Dorothea Emig, Alexander Ivliev, Olga Pustovalova, Lee Lancashire, Svetlana Bureeva, Yuri Nikolsy, Marina Bessarabova

Thomson Reuters, 5901 Priestly Dr, Suite 200, Carlsbad, CA 92008, USA

In the following, the ROC plots with 95% confidence intervals of the drug target predictions for all 30 diseases are shown.


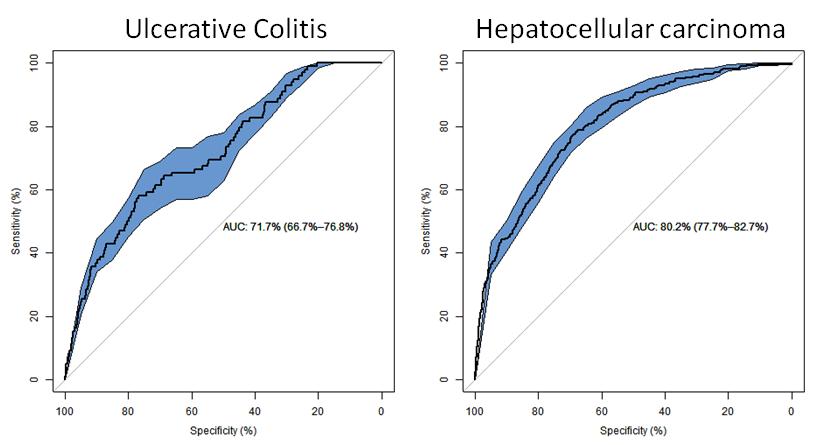


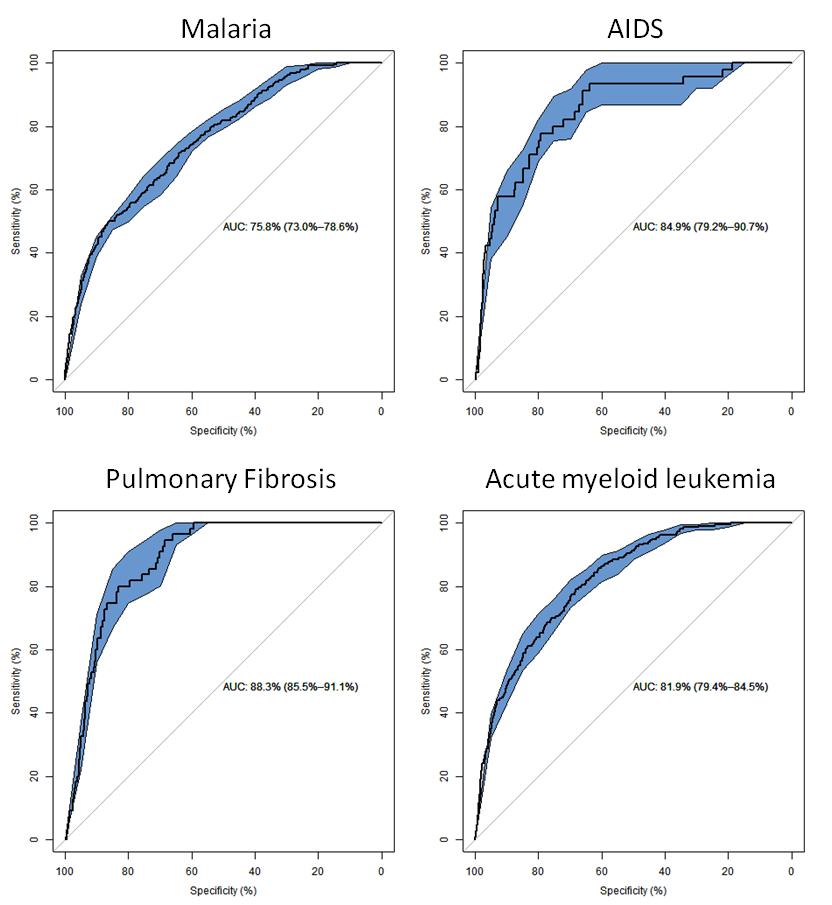


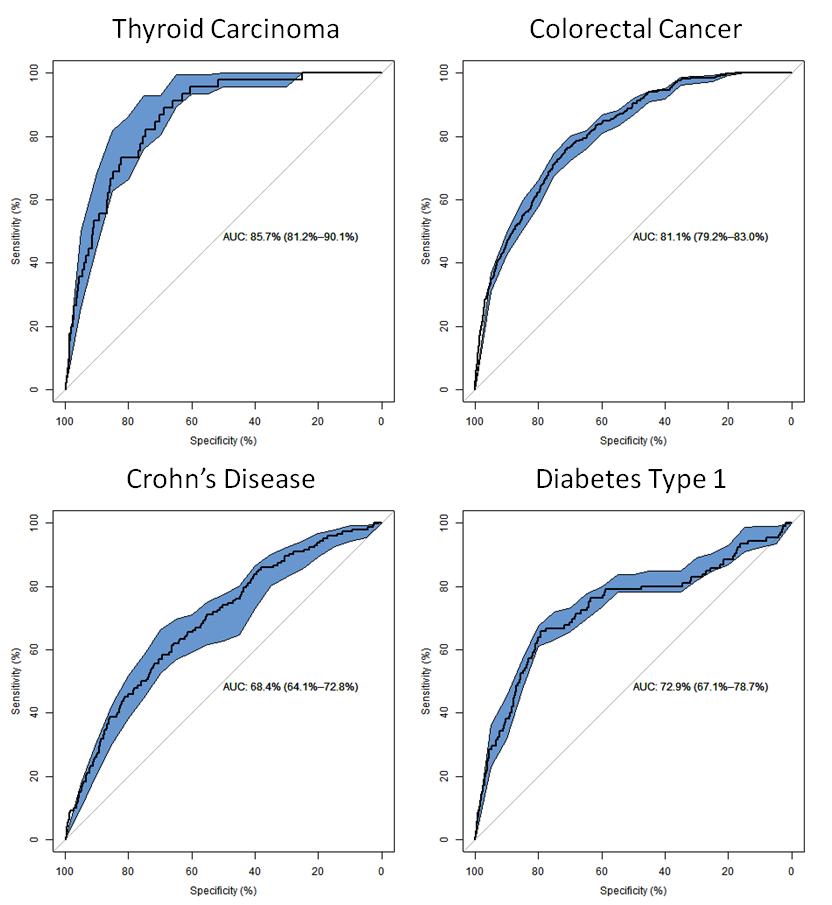


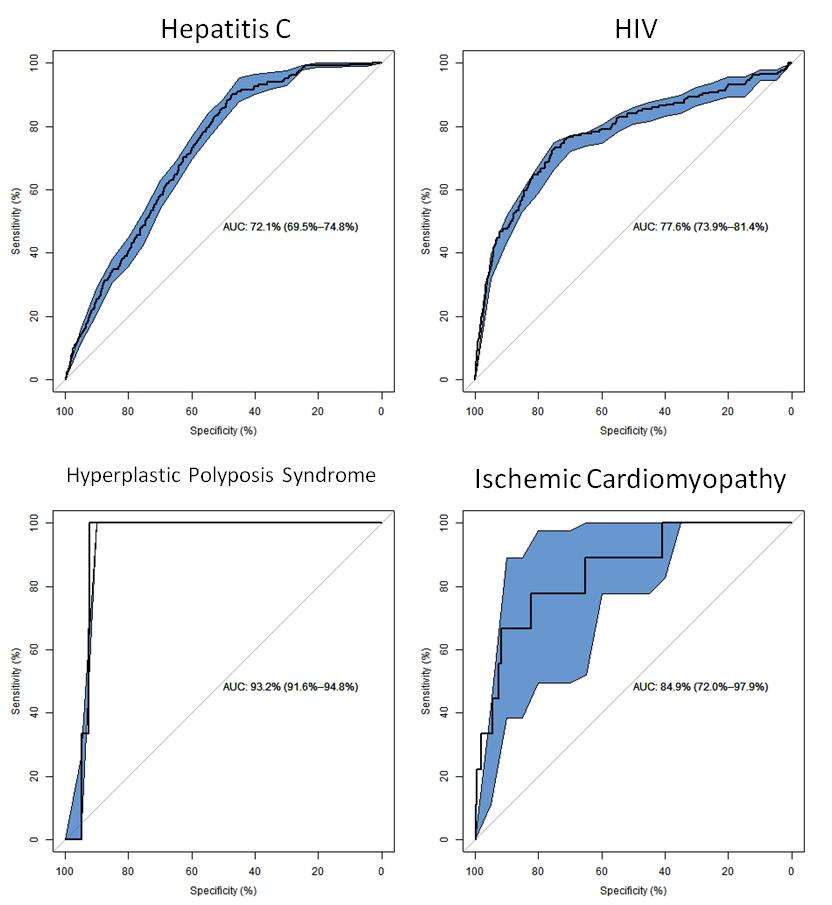


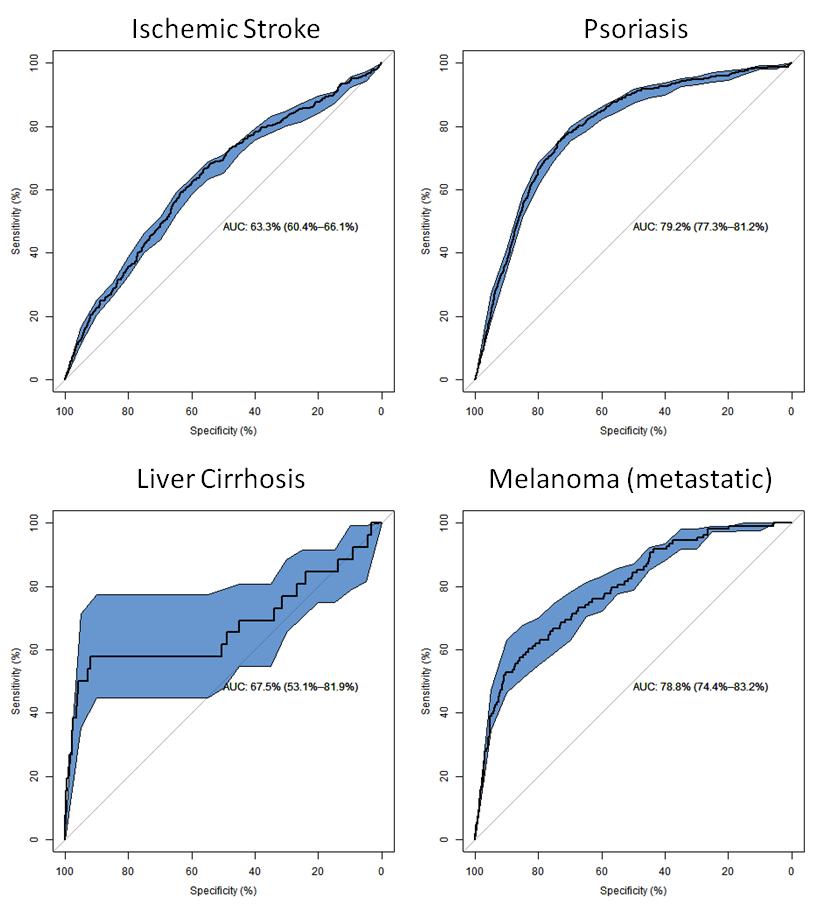


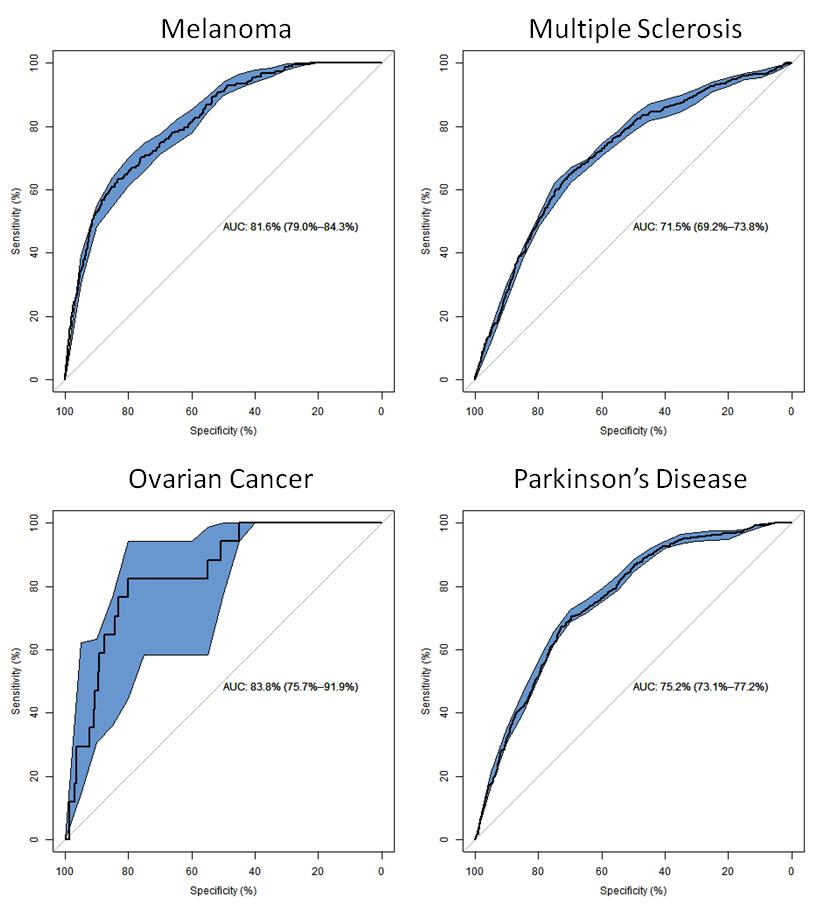


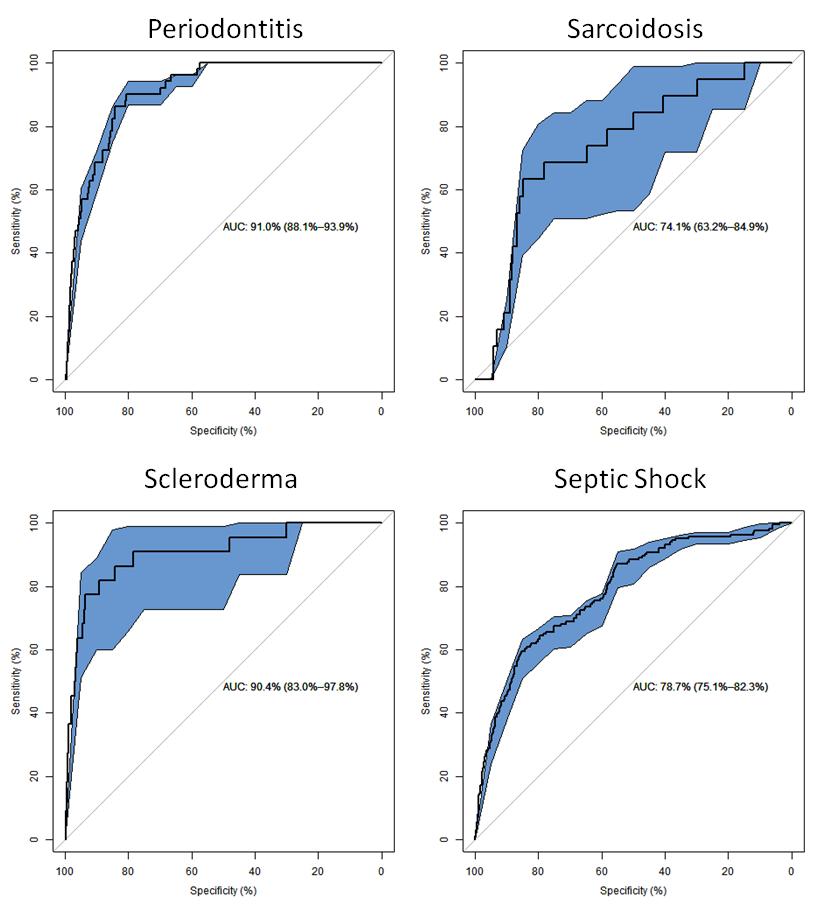


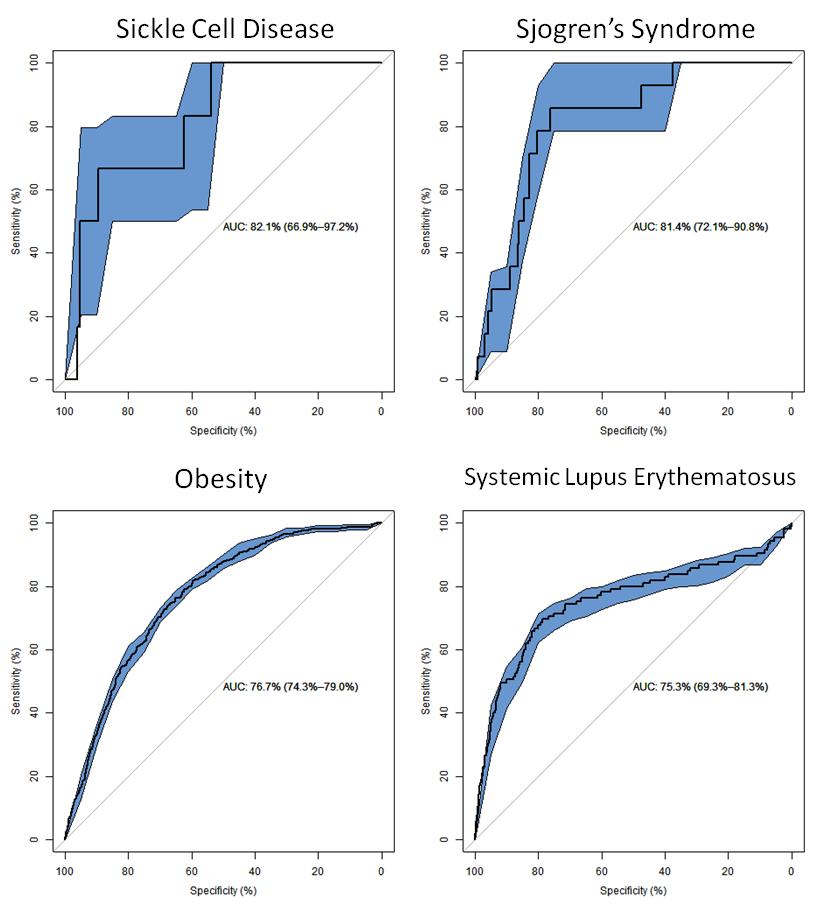

Supplement: File S1 — Prediction performance for 30 diseases. The file contains the ROC plots for all 30 diseases. The blue area around each curve represents the 95% confidence interval. (DOC) [file pone.0060618.s001.doc]
